# Insufficient radiofrequency ablation promotes human hepatoma SMMC7721 cell proliferation by stimulating vascular endothelial growth factor overexpression

**DOI:** 10.3892/ol.2015.2966

**Published:** 2015-02-16

**Authors:** ZHINING LIU, HONGLIANG DAI, GUIZHI JIA, YUHONG LI, XIN LIU, WEIDONG REN

**Affiliations:** 1 Ultrasound Department, Shengjing Hospital, China Medical University, Shenyang, Liaoning 110004, P.R. China; 2 School of Nursing, Liaoning Medical University, Jinzhou, Liaoning 121001, P.R. China; 3 Department of Biochemistry and Molecular Biology, Liaoning Medical University, Jinzhou, Liaoning 121001, P.R. China; 4 Ultrasound Department, First Affiliated Hospital, Liaoning Medical University, Jinzhou, Liaoning 121001, P.R. China; 5 Clinical Laboratory, First Affiliated Hospital, Liaoning Medical University, Jinzhou, Liaoning 121001, P.R. China

**Keywords:** Ca^2+^/calmodulin-dependent protein kinase II, extracellular signal-regulated kinase, radiofrequency, SMMC7721

## Abstract

The aims of the current study were to investigate the influence of insufficient radiofrequency ablation (RFA) on the cell proliferation of the human hepatocellular carcinoma (HCC) cells, SMMC7721, and to elucidate the underlying mechanism. SMMC7721 cells were subjected to a 47°C treatment regimen to simulate insufficient RFA, in the presence or absence of KN93 [a specific inhibitor of Ca^2+^/calmodulin-dependent protein kinase II (CaMKII)], PD98059 [a specific inhibitor of extracellular signal-regulated kinase (ERK)], or axitinib (a specific inhibitor of vascular endothelial growth factor (VEGF) receptor]. Cell proliferation was determined using a thiazolyl terazolium assay (MTT). The levels of CaMKII, phospho-CaMKII, ERK, phospho-ERK and VEGF were observed by western blot analysis. The results demonstrated that the 47°C treatment regimen: i) Triggered upregulation of VEGF expression in the SMMC7721 cells, which was reduced by CaMKII or ERK inhibition; ii) induced ERK activation was prevented by KN93; and iii) promoted SMMC7721 cell proliferation, which was greatly inhibited by axitinib, KN93 and PD98059. In conclusion, the results indicated that insufficient RFA promotes SMMC7721 cell proliferation by activating CaMKII/ERK-dependent VEGF overexpression.

## Introduction

Hepatocellular carcinoma (HCC) is the most common primary liver tumor and a major public health problem worldwide (1), representing the third most common cause of cancer-associated mortality (2). Although hepatic resection and transplantation have been regarded as the optimum therapeutic strategies, in a large number of cases, certain limitations, including lack of donors, severity of liver disease and specific selection criteria (Milan criteria), restrict the application of these strategies (3,4). Consequently, these surgical methods are appropriate in only ~30% of HCC cases (5). At present, locoregional treatments, such as image-guided radiofrequency ablation (RFA), are promising in the treatment of various carcinoma types, including HCC (6). As a novel treatment strategy, RFA possesses multiple advantages, including the capacity for localized tumor necrosis, minimal damage to functioning liver and the capacity for repeated treatments in case of recurrence and/or new tumors (7). However, previous studies have proposed that insufficient RFA may promote the proliferation of residual HCC (8,9). Therefore, elucidating the molecular mechanisms underlying this undesirable effect of RFA on HCC is important.

Angiogenesis is one of the critical mechanisms involved in the proliferation and growth of tumor cells (10). Among the numerous angiogenesis-associated genes, vascular endothelial growth factor (VEGF) functions as a potent angiogenic factor and is ubiquitously expressed in various types of cancer, including HCC (9,11). A previous study has demonstrated that VEGF overexpression in HCC is typically associated with tumor progression, reduced median survival and recurrence following treatment (12). In addition, a recent study has identified that insufficient RFA induced the aggressive growth of residual HCC mediated by VEGF overexpression (9). However, the underlying mechanisms by which insufficient RFA enhances VEGF expression remain unknown. The aim of the present study was to investigate the mechanisms by which insufficient RFA promotes HCC proliferation, particularly the intracellular signaling pathway(s) involved in VEGF overexpression.

## Materials and methods

### Cell culture

The established human HCC cell line, SMMC7721, was obtained from the American Type Culture Collection (Manassas, VA, USA). The cells were maintained in RPMI-1640 (Corning Inc., Corning, NY, USA) supplemented with 10% fetal bovine serum (Hyclone-Thermo Fisher Scientific, Waltham, MA, USA) in a humidified atmosphere of 5% CO_2_ at 37°C.

### Heat treatment

Insufficient RFA was simulated *in vitro* using a previously described method (13). Briefly, SMMC7721 cells were seeded into 6-well plates (5×10^4^ cells/well). After 24 h, the plates were sealed and submerged in a water bath at 47°C for 5 min. Subsequently, the cultures were cultured in RPMI-1640 medium supplemented with 10% fetal bovine serum in a humidified atmosphere of 5% CO_2_ at 37°C until residual populations reached 80% confluence. The surviving populations were propagated into the 6-well plates and exposed to the aforementioned heat treatment for 10 min. Subsequently, this process was repeated with the stimulation time increasing with each repetition (15, 20 and then 25 min). Cells surviving the 47°C treatment regimen were designated as the ‘47°C treatment’ group and used in subsequent experiments. SMMC7721 cells that were not exposed to the 47°C treatment regimen were used as the ‘control’ group. To investigate the effect of Ca^2+^/calmodulin-dependent protein kinase II (CaMKII), extracellular signal-regulated kinase (ERK) and VEGF on the growth of residual SMMC7721 cells in the 47°C treatment group, 10 μM KN93, a specific CaMKII inhibitor, 20 μM PD98059, a specific ERK inhibitor, or 5 μM axitinib, a VEGF receptor antagonist (all purchased from Sigma-Aldrich, St. Louis, MO, USA), was added to cell cultures obtained from the ‘control’ group, termed the ‘KN93’, ‘PD98059’ and ‘axitinib’ groups, respectively, and/or the ‘47°C treatment’ group, termed the ‘KN93 + 47°C treatment’, ‘PD98059 + 47°C treatment’ and ‘axitinib + 47°C treatment’ groups, respectively.

### Proliferation assay

Cell proliferation was analyzed using an MTT assay [3-(4,5-dimethylthiazol-2-yl)-2,5-diphenyltetrazolium bromide]. Briefly, a total of 3×10^3^ trypsin-dispersed parent SMMC7721 cells or 47°C-treated SMMC7721 cells in 0.1 ml culture medium were seeded into 96-well plates and cultured for 24, 48 and 72 h. MTT solution was added to each well at a final concentration of 0.5 mg/ml and incubated for 4 h. At the end of the incubation, formazan crystals resulting from the MTT reduction assay were dissolved through the addition of 150 μl dimethyl sulfoxide per well. The absorbance was measured at 570 nm using an automated ELISA plate reader (Thermo Fisher Scientific, Waltham, MA, USA).

### Western blot analysis

Cells grown in culture dishes were washed with phosphate-buffered saline (Solarbio Science & Technology Co., Ltd, Beijing, China) and harvested using a cell scraper (Nest Biotechnology Co., Ltd, Wuxi, China). Whole-cell lysates were collected by adding freshly prepared lysis buffer [containing 150 mM NaCl, 50 mM Tris-HCl, pH 8.0, 0.1% sodium dodecyl sulfate, 1% Triton X-100 and 1% proteinase inhibitors (1:100, Sigma-Aldrich)] to the harvested cells, which were then incubated on ice for 30 min. Next, the cell lysates were centrifuged at 14,000 × g for 20 min at 4°C (Sorvall™ ST 16R, Thermo Fisher Scientific) and the protein content was determined by Lowry’s method (14) using bovine serum albumin as the standard. The samples were adjusted to contain 30 μg protein each; subsequently, they were subjected to SDS-PAGE and electrophoretically transferred to a polyvinylidene fluoride membrane. Following transfer, the membrane was blocked with 5% non-fat milk for 1 h at room temperature then incubated overnight at 4°C with primary rabbit anti-human polyclonal anti-VEGF (1:200; cat. no. BA0407; Wuhan Boster Biological Technology, Ltd., Wuhan, China), anti-CaMKII (1:500; cat. no. sc-13082; Santa Cruz Biotechnology, Inc., Dallas, TX, USA), anti-phospho-CaMKII (1:1,000; cat. no. V1111; Promega Corporation, Madison, WI, USA), anti-ERK (1:1,000; cat. no. sc-292838; Santa Cruz Biotechnology, Inc.) or anti-phospho-ERK (1:1,000; cat. no. sc-101760; Santa Cruz Biotechnology, Inc.) antibodies. Subsequently, the membrane was incubated with polyclonal horseradish peroxidase conjugated-secondary goat anti-rabbit antibodies (1:3,000; cat. no. sc-2004; Santa Cruz Biotechnology, Inc.) diluted in Tris-buffered saline and Tween-20 (containing 20 mM Tris-HCl, 150 mM NaCl, pH 7.4 and 0.1% Tween-20) for 2 h at room temperature. Finally, the membrane was rinsed with phosphate-buffered saline and visualized using an enhanced chemiluminescence detection kit (Thermo Fisher Scientific). The western blot bands were quantified using QuantityOne software (version 4.6.9; Bio-Rad Laboratories, Inc., Hercules, CA, USA) by measuring the band intensity for each group and normalizing against β-actin, which was used as an internal control.

### Statistical analysis

Data are expressed as the mean ± standard error of mean from at least three independent experiments. Statistical analysis for multiple comparisons was performed using one-way analysis of variance followed by Fisher’s least significant difference test. All statistical analyses were performed using SPSS version 17.0 software (SPSS, Inc., Chicago, IL, USA). Differences were considered to be statistically significant when P<0.05.

## Results

### VEGF overexpression is triggered by 47°C heat treatment and blocked by ERK and CaMKII

**Figure 1:**
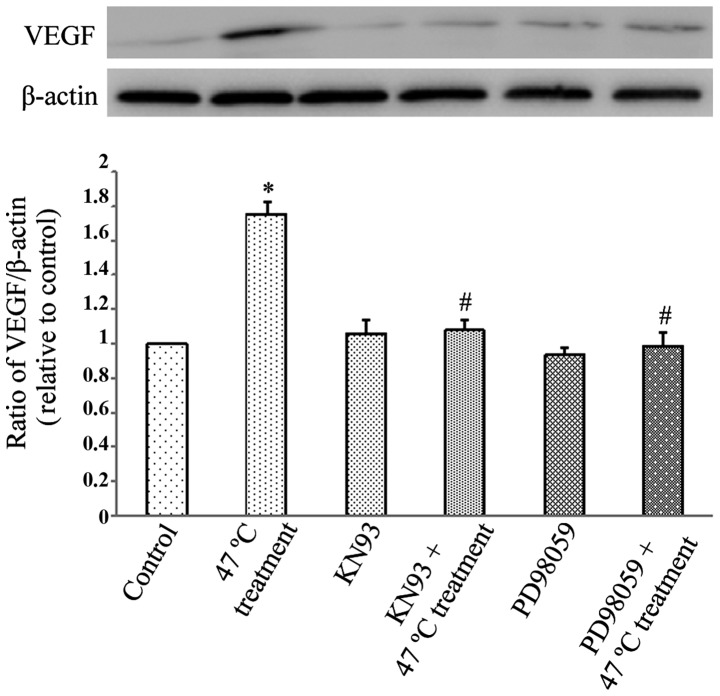
VEGF expression increased following 47°C treatment and is ERK- and CaMKII-dependent. Data are expressed as the mean ± standard error of mean from four independent experiments. ^*^P<0.05, vs. control group. ^#^P<0.05, vs. 47°C treatment group. VEGF, vascular endothelial growth factor; ERK, extracellular signal-regulated kinase; CaMKII, Ca^2+^/calmodulin-dependent protein kinase II.

To investigate how insufficient RFA triggers VEGF overexpression, specific pharmacological inhibitors of ERK (PD98059) and CaMKII (KN93) were used. Fig. 1 demonstrates that the 47°C treatment regimen significantly upregulated VEGF protein expression in SMMC7721 cells (P<0.05), and this effect was significantly reduced by pretreatment with PD98059 and KN93 (P<0.05). Therefore, this experiment demonstrated that the 47°C treatment regimen induced VEGF overexpression through the activation of ERK and CaMKII signaling pathways.

### Interaction between CaMKII and ERK

**Figure 2:**
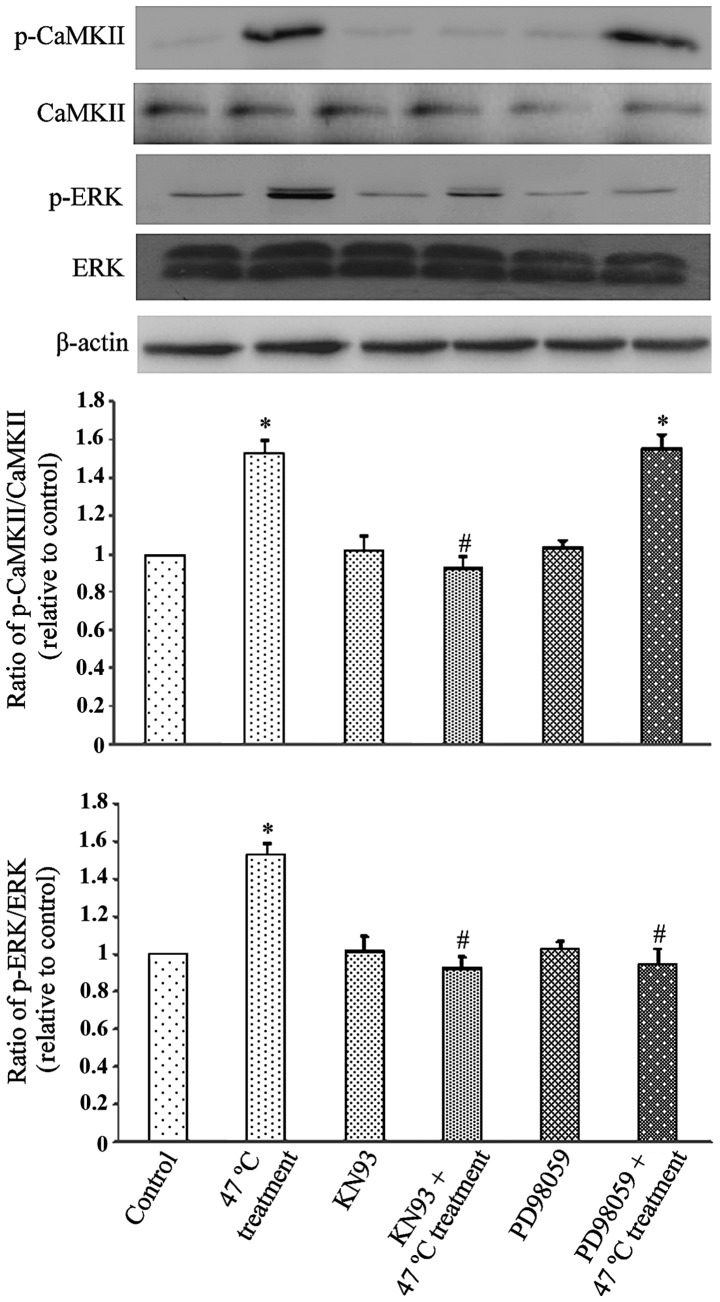
CaMKII phosporylation occurs upstream of ERK phosporylation during 47°C treatment. Data are expressed as the mean ± standard error of mean from four independent experiments. ^*^P<0.05 vs. control group. ^#^P<0.05 vs. 47°C treatment group. VEGF, vascular endothelial growth factor; ERK, extracellular signal-regulated kinase; CaMKII, Ca^2+^/calmodulin-dependent protein kinase II; p, phosphorylated.

The possible interaction between CaMKII and ERK following the 47°C treatment was investigated. As Fig. 2 demonstrates, the 47°C treatment triggered a significant increase in ERK activation (phosphorylation; P<0.05), which was inhibited by PD98059 and KN93 (P<0.05). The 47°C treatment also triggered a significant increase in CaMKII phosphorylation (P<0.05), which was only blocked by KN93 (P<0.05) and not by the inhibitor of ERK, PD98059. These observations indicate that CaMKII activation is required for ERK activation following the 47°C heat treatment, but not vice versa.

### Effect of PD98059, KN93 and axitinib on SMMC7721 cell proliferation

**Figure 3:**
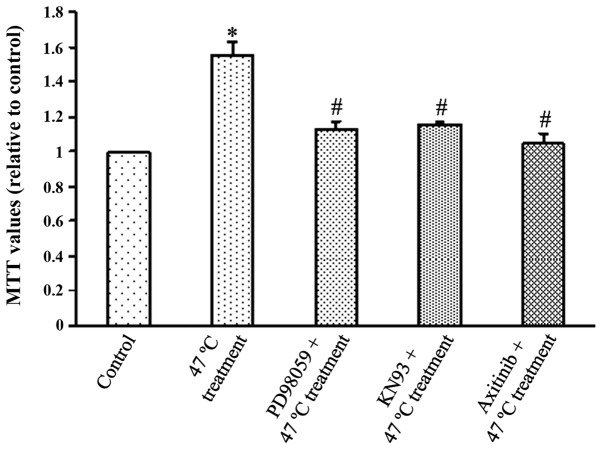
Proliferation of SMMC7721 cells following 47°C treatment is dependent on CaMKII (inhibitor, KN93), ERK (inhibitor, PD98059) and VEGFR (inhibitor, axitinib). Data are expressed as the mean ± standard error of mean from five independent experiments. ^*^P<0.05, vs. control. ^#^P<0.05, vs. 47°C treatment group. VEGFR, vascular endothelial growth factor receptor; ERK, extracellular signal-regulated kinase; CaMKII, Ca^2+^/calmodulin-dependent protein kinase II.

Fig. 3 demonstrates that SMMC7721 cells in the 47°C treatment group exhibited significantly higher viability compared with the control group at 48 h after the 47°C treatment (P<0.05), indicating that the 47°C heat stimulation increased cell proliferation. Notably, PD98059 or KN93 pretreatment inhibited this effect (P<0.05), indicating that ERK and CaMKII may act to increase tumour cell proliferation. Axitinib, a VEGF receptor (VEGFR) antagonist, also inhibited SMMC7721 cell viability (P<0.05), demonstrating that elevated VEGF may exert its growth promoting effects through VEGFR.

## Discussion

As a result of its multiple advantages, RFA is currently widely used for HCC treatment as an alternative to traditional surgery. However, mounting clinical and experimental evidence has revealed that RFA treatment may cause rapid growth of any residual HCC (8,15). However, the underlying mechanisms and factors that mediate this rapid growth of residual HCC following RFA remain unclear. Several mediators have been proposed to be involved in this undesirable process, including increased VEGFA expression (9). VEGFA is the most important angiogenic molecule of the VEGF family, which includes VEGFA, VEGFB, VEGFC, VEGFD and placental growth factor (PLGF) (9). The present study did not differentiate between the VEGF subtypes, but identified a similar enhanced expression of total VEGF following a 47°C treatment regimen, which was used to simulate RFA. In addition, the specific VEGFR inhibitor, axitinib (16), was found to greatly suppress 47°C heat stimulated tumor cell proliferation. Therefore, VEGF upregulation that was triggered by the heat stimulation exerted its proliferative effect by binding to and activating its receptor, VEGFR.

Subsequently, the underlying mechanism of this enhanced VEGF expression was investigated. The Raf/MEK/ERK signaling pathway is downstream of Ras activation, and tyrosine phosphorylation of these signaling molecules is essential to cancer cell proliferation (17). A recent study identified that HCC exhibited increased expression of phospho-ERK and enhanced proliferation following insufficient RFA (13). In addition, the specific ERK inhibitor, PD98059, significantly suppressed the malignancy of HCC in a previous study (13), as well as in the present study, demonstrating a critical role of ERK in insufficient RFA-induced HCC malignancy. The present study, demonstrated that VEGF overexpression following the 47°C treatment regimen was significantly downregulated by PD98059, demonstrating that upregulated VEGF expression by heat stimulation was ERK-dependent.

CaMKII is a ubiquitous mediator of Ca^2+^-associated signaling that phosporylates various substrates to coordinate and regulate Ca^2+^-mediated modifications in cellular function (18). ERK is one of the targets of CaMKII and the CaMKII-ERK cascade has been identified in a recent study (19). Furthermore, a previous study demonstrated that the CaMKII-mediated activation of ERK contributed to cell proliferation of papillary thyroid carcinoma (20). Based on these observations, the present study hypothesized that the ERK activation triggered by the 47°C heat treatment may be CaMKII-dependent. ERK phosphorylation was inhibited by PD98059 (an ERK inhibitor) and KN93 (a specific inhibitor of CaMKII); however, PD98059 did not affect CaMKII phosphorylation. CaMKII may operate upstream of ERK, as this study initially hypothesized. Furthermore, the present study confirmed that upregulated VEGF expression and tumor cell proliferation were significantly decreased by KN93.

In conclusion, the present study described a possible mechanism of the growth promoting effects of RFA on residual HCC. RFA promoted residual HCC growth through increasing VEGF expression via CaMKII-induced ERK activation. These results may improve the understanding of the mechanisms of residual HCC progression and relapse, and thus provide novel, effective targets for the prevention and treatment of this undesirable effect during RFA therapy.
